# Novel functional small RNAs are selectively loaded onto mammalian Ago1

**DOI:** 10.1093/nar/gku137

**Published:** 2014-03-13

**Authors:** Natsuko Yamakawa, Kazuki Okuyama, Jun Ogata, Akinori Kanai, Aleksandra Helwak, Masako Takamatsu, Ken-ichi Imadome, Kohei Takakura, Bidisha Chanda, Natsumi Kurosaki, Haruna Yamamoto, Kiyoshi Ando, Hirotaka Matsui, Toshiya Inaba, Ai Kotani

**Affiliations:** ^1^Department of Regenerative Medicine, Division of Basic Clinical Science, Tokai University School of Medicine, Isehara, Kanagawa 259-1193, Japan, ^2^Department of Molecular Oncology and Leukemia Program Project, Research Institute for Radiation Biology and Medicine, Hiroshima University, Hiroshima 734-8553, Japan, ^3^Wellcome Trust Centre for Cell Biology, The University of Edinburgh, Edinburgh, EH9 3JR, UK, ^4^Department of Infectious Diseases, National Research Institute for Child Health and Development, Tokyo 157-0074, Japan, ^5^Department of Biosciences, School of Science, Kitasato University, Kanagawa 252-0373, Japan, ^6^Research Center for Regenerative Medicine, Tokai University School of Medicine, Kanagawa 259-1193, Japan and ^7^Precursory Research for Embryonic Science and Technology (PRESTO), Japan Science and Technology Agency (JST), Saitama 332-0012, Japan

## Abstract

Argonaute (Ago) proteins function in RNA silencing as components of the RNA-induced silencing complex (RISC). In lower organisms, the small interfering RNA and miRNA pathways diverge due in part to sorting mechanisms that direct distinct small RNA (sRNA) duplexes onto specific Ago-RISCs. However, such sorting mechanisms appear to be lost in mammals. miRNAs appear not to distinguish among Ago1–4. To determine the effect of viral infection on the sorting system, we compared the content of deep-sequenced RNA extracted from immunoprecipitation experiments with the Ago1 and Ago2 proteins using Epstein–Barr virus (EBV)-infected cells. Consistent with previous observations, sequence tags derived from miRNA loci in EBV and humans globally associate in approximately equivalent amounts with Ago1 and Ago2. Interestingly, additional sRNAs, which have not been registered as miRNAs, were associated with Ago1. Among them, some unique sequence tags derived from tandem loci in the human genome associate exclusively with Ago1 but not, or rarely, with Ago2. This is supported by the observation that the expression of the unique sRNAs in the cells is highly dependent on Ago1 proteins. When we knocked down Ago1, the expression of the Ago1-specific sRNAs decreased dramatically. Most importantly, the Ago1-specific sRNAs bound to mRNAs and regulated target genes and were dramatically upregulated, depending on the EBV life cycle. Therefore, even in mammals, the sorting mechanism in the Ago1–4 family is functional. Moreover, the existence of Ago1-specific sRNAs implies vital roles in some aspects of mammalian biology.

## INTRODUCTION

miRNAs are a recently discovered class of small noncoding RNAs that are 18–24 nucleotides long and that downregulate target genes at the posttranscriptional level. The majority of miRNA genes are transcribed by RNA polymerase II into long primary miRNA transcripts, processed by the nuclear nuclease Drosha into ∼60-bp hairpins, termed precursor (pre) miRNAs, and further cleaved in the cytosol by Dicer nuclease into mature miRNAs. Mature miRNAs are then incorporated into the multiprotein RNA-induced silencing complex (RISC), exerting posttranscriptional repression of target mRNAs, either by inducing mRNA cleavage and mRNA degradation or by blocking mRNA translation (1).

RNAi plays a critical role in innate cellular defence against viruses. In plants and invertebrates, viral RNA genomes and mRNAs are targeted for destruction by the stimulated production of small interfering RNA (siRNAs) derived from viral double-stranded RNAs (2,3). Given the strong type I interferon-based antiviral response initiated by the intracellular double-stranded RNA sensors RNA-activated protein kinase (PKR), Retinoic acid-inducible gene-I (RIG-I) and Melanoma differentiation-associated gene 5 (MDA-5) in mammals, the siRNAi pathway may have been suppressed as a host protection mechanism against RNA viruses (4). Recently, analogous viral siRNAs were detected within the pool of small RNAs (sRNAs) isolated from RNA-virus–infected mammalian Embryonic stem (ES) cells that exhibited a low-level interferon-based antiviral response.

In lower organisms, the siRNA and miRNA pathways diverge in part due to sorting mechanisms that direct distinct sRNA duplexes into specific Argonaute (Ago)-RISCs (5). However, such sorting mechanisms appear to be lost in mammals. miRNAs appear not to distinguish among Ago1–4, while only Ago1 and Ago2 prefer siRNAs (6). In contrast, influenza A virus infected Ago 1 and 3 double-knockout mice exhibited increased mortality, consistent with more severe alveolitis and pneumonitis, indicating that optimal resistance to influenza requires Ago 1 and/or 3. Enhanced mortality of double-knockout mice was not associated either with increased viral replication or with differential pulmonary recruitment or function of innate and adaptive immune cells; therefore, its function in RNAi targeting against virus-coding RNAs has yet to be demonstrated. The results show that while miRNAs may not distinguish among Ago1–4, this may not be the case for other sRNAs (7). Epstein–Barr virus (EBV), a member of the γ-herpes virus family, was found to be widespread in all human populations and to persist in the vast majority of individuals as a lifelong, asymptomatic infection of the B-lymphocyte pool. It is usually the cause of infections that are not apparent, though it may cause infectious mononucleosis. The more severe, albeit rare, result of EBV infection is malignant transformation and cancer development in various forms, including Burkitt’s lymphoma and nasopharyngeal carcinoma, one of the most common cancers in China. As a ubiquitous human pathogen, EBV-associated lymphoid malignancies include a subset of Burkitt’s lymphoma, AIDS lymphoma, Hodgkin’s lymphoma, posttransplant lymphoma, age-associated B-cell lymphoma and peripheral T- and NK-cell lymphoma (8).

Like other herpes viruses, EBV infection can exhibit two distinct patterns or states of gene expression. During acute EBV infection, the virus sequentially expresses its entire repertoire of genes, producing a lytic infection. In this lytic program, linear double-stranded genomes are produced and packaged as virions that spread the infection from cell to cell. In the latent program, few viral genes are transcribed, no viral progeny are produced and infected cells are protected from apoptotic stimuli and in some circumstances driven to proliferate (8).

EBV was the first human virus found to encode micro RNAs. EBV encodes 44 viral micro RNAs and one small-RNA. EBV-encoded micro RNAs originated from the Bam HI fragment H rightward open reading frame 1 (BHRF1) and Bam HI A region rightward transcript (BART) loci of the EB viral genome. These viral micro RNAs play a vital role in immunogenesis, host cell survival and proliferation, differentiation, lymphomagenesis and modulating the states of viral infection and latency (8).

To determine whether the viral-encoded RNAs are selectively sorted and if the sorting system is affected by viral infection, we compared the content of deep-sequenced RNA extracted from immunoprecipitation (IP) experiments with the Ago1 and Ago2 proteins using EBV-infected cells. The EBV-encoded miRNAs are incorporated equally into Ago1 and Ago2 and identified a novel class of sRNAs that are preferentially incorporated into Ago1 but not Ago2. In this study, we investigated the abundance, expression patterns, sequence characteristics and functions of the novel Ago1-specific–associated sRNAs.

## MATERIALS AND METHODS

### Cells

The EBV-positive and -negative Hodgkin’s lymphoma cell lines, L591 and L1236 (9), and Akata (+) cells and acute monocytic leukemia cell lines, THP-1, were maintained in RPMI1640 medium (Nacalai tesque) supplemented with 10% (v/v) fetal bovine serum (FBS), 50 U/ml penicillin and 50 µg/ml streptomycin in a 50-ml flask (Sumilon). Human carcinoma of cervix cell line, HeLa, was maintained in Dulbecco's modified Eagle's medium (Nacalai tesque) supplemented with 10% (v/v) FBS, 50 U/ml penicillin and 50 µg/ml streptomycin in a 10-cm dish (Corning, Inc., Corning, NY, USA). Cells were passaged twice per week.

### Immunoprecipitation

Small RNAs incorporated into Ago1 and Ago2 were immunoprecipitated from cell lysates using the microRNA Isolation Kit (human/mouse Ago1 and human Ago2) (Wako, Osaka, Japan) according to the manufacturer’s protocol.

### Next-generation sequencing

Immunoprecipitated sRNAs were converted to sequence libraries using the sRNA sample preparation kit (Illumina), according to the manufacturer’s instructions.

The libraries were sequenced using an Illumina GA IIx with the single 36-bp read option. Adapter sequences were removed from the generated sequence data. The adapter-trimmed 20–24-bp sequences were mapped to the human hg19 genome assembly and EBV genome using the sequence alignment software Eland (Illumina).

Mapped sequences without any mismatches were used for further analysis. Redundantly mapped sequences that were <256 positions in the genomes were counted as 1 sequence at all possible mapped positions.

### siRNA and transfection

siRNA targeting human *Ago1* and *DROSHA* was purchased from OriGene Technology, Inc. (MD, USA) and Cosmo Bio Co., Ltd. (Tokyo, Japan), respectively. siRNA against human *Ago2* was kindly provided by Dr N. Kosaka (National Cancer Center Research Institute, Tokyo, Japan). L591 cells, L1236 cells and Akata cells were suspended in resuspension buffer at a density of 2 × 10^7^/ml and siRNA or the negative control synthetic RNA (Bioneer, Inc., CA, USA) was added at a final concentration of 1 µM (for Akata cells, 500 nM). The transfection of siRNA into cells was performed with the Neon® transfection system (Invitrogen, co., CA, USA) according to the manufacturer’s protocol. Cells were cultured for 3 or 6 days in RPMI1640 medium supplemented with 10% (v/v) FBS.

Sequences of specific siRNAs were described in Supplementary Table S1.

### Quantitative polymerase chain reaction for Agotaxis sRNAs

Total RNA was prepared from cells with Sepasol-RNA I Super G (Nakarai Tesque, Inc., Kyoto, Japan) and reverse-transcribed with the miScript II RT Kit (Qiagen, Hilden, Germany) to synthesize complementary DNA of sRNAs. The sRNAs were polyadenylated by poly(A) polymerase, then, bound by oligo-dT primer that have a 5′ universal tag sequence and 3′ degenerate anchor and reverse-transcribed by miScript Reverse Transcriptase. The cDNA with the universal tag was amplified by its complementary sequence and the gene-specific forward primers. Real-time polymerase chain reaction (PCR) was performed using the StepOne software and miScript SYBR Green PCR Kit (QIAGEN). Each of 2×SYBR Master Mix 5 µl, RNase-free water 2 µl, 5 µM target primer 1 µl and 1 µl of 10× universal primer mixture were added to 1 µl of water-soluble sample of cDNA. The reaction was run at 95°C for 10 min, followed by 40 cycles at 95°C for 15 s and 60°C for 1 min. miR-21, miR-155, RNU6 and candidate sRNAs were detected by quantitative PCR (qPCR) using the miScript SYBR Green PCR Kit (Qiagen). Sequences of specific primers were described in Supplementary Table S1.

### Quantitative PCR for genes

For target gene detection, RT-PCR was performed using the High Capacity cDNA Reverse Transcription Kit (Applied Biosystems, Inc., CA, USA) and qPCR was carried out with the Fast SYBR Green Master mix. All real-time qPCR was conducted using the StepOnePlus real-time PCR system (Applied Biosystems). Threshold cycle (CT) values were calibrated to Glyceraldehyde-3-phosphate dehydrogenase (GAPDH) and analyzed by the 2-ΔΔCT method. Statistical analysis was done by student t-test. Sequences of specific primers were described in Supplementary Table S1.

### Dual luciferase assay

In each well of a 96-well plate, 293T cells were cotransfected with 30 ng of psiCHECK-2 s (Promega) and 90 ng of siRNA. The siRNAs were purchased from Cosmo Bio Co., Ltd. (Tokyo, Japan). Sequences of the siRNAs for Dual luciferase assay were described in Supplementary Table S1.

After 48 h of transfection, the relative amounts of Renilla and firefly luciferase were determined by dual-luciferase assay (Promega). The Renilla/firefly luciferase ratio was calculated and normalized against the control.

### Gene expression analysis

RNA from cells used for microarray analysis was isolated using the RNeasy Mini Kit (Qiagen, Hilden, Germany). For microarray analysis, splenocytes were cultured for 72 h with or without 10 µM IM. Gene expression microarray analysis was performed using two-colour microarray-based gene-expression analysis (Agilent Technologies, Santa Clara, CA, USA) according to the manufacturer’s instructions. After scanning, expression values for the genes were determined using GeneSpringGX software. All experiments were performed in duplicate.

### Analysis of CLASH data

Sequencing data generated with the CLASH technique and recently published (ref., GEO accession GSM1219490) were analyzed as previously reported (10) with the following modifications:

1. Custom BLAST database was supplemented with the sequences of Agotaxis sRNAs (ASR1: gagaaagctcacaagaactgct; ASR2: ccccccactgctaaatttgactg; ASR3: tcccactgcttcacttgactagc; ASR4: tcccccactgctaaatttgactgg; ASR5: aagcagggtcgggcctggt; ASR6: gggaataccgggtgctgtaggc).
2. We selected all chimeras such that one bit was mapped to an ASR and the other bit was uniquely assigned to a protein coding gene (multiple transcripts allowed). Overview diagrams of ASRs-mRNA chimera preparation are presented in Figure 5b.

### Lytic phase induction assay

Akata (+) cells were stimulated with rabbit anti-human IgG polyclonal antibody (final 20 μg/ml) (Dako) at 37°C in a CO_2_ incubator for 15–40 h and induced into lytic phase. Cells were counted before and after stimulation and then the numbers of dead cells were calculated. For Ago1 knocked down experiments, siRNAs were electroporated into the cells by Neon® transfection system 2 days before anti-human IgG stimulation and the cells were cultured for 48 h. After stimulation, cells were harvested and total RNAs were prepared with Sepasol-RNA I Super G. To quantify sRNA expression, RT-PCR was performed using the miScript II RT kit, and qPCR using the miScript SYBR Green PCR kit. For target gene detection, RT-PCR was performed using the high-capacity RNA-to-cDNA kit and qPCR was carried out with the Fast SYBR Green Master mix. All real-time qPCR was conducted using the StepOnePlus real-time PCR system.

## RESULTS

### Ago-associated sRNA libraries indicate that equivalent amounts of human and EBV-encoded miRNAs associated with Ago1 and Ago2

We analyzed the short RNAs from immunoprecipitated Ago1 and Ago2 proteins isolated from L591cells derived from EBV-positive Hodgkin’s lymphoma cells. Raw tags from the generated data set were mapped to the hg19 human genome assembly using the procedures outlined in the ‘Materials and Methods’ section.

**Figure 1.**
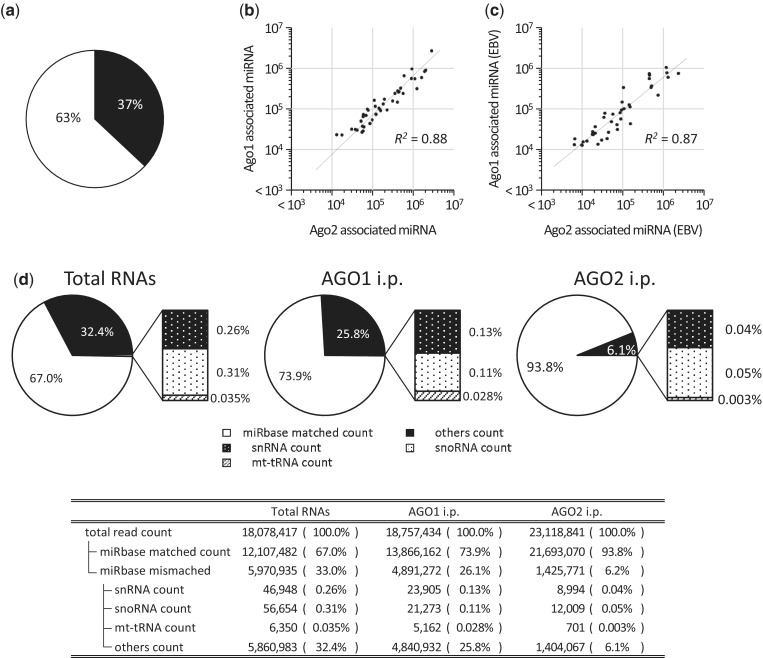
Equivalent amounts of human and EBV-encoded miRNAs associated with Ago1 and Ago2. The short RNAs that were co-immunoprecipitated with Ago1 and Ago2 derived from L591 cells were analyzed. (**a**) The ratio of sRNAs mapped to the human genome and the EBV genome. (**b**) Equivalent amounts of human miRNAs associated with Ago1 and Ago2. The top 50 highly associated human miRNAs were plotted. (**c**) Equivalent amounts of EBV-encoded miRNAs associated with Ago1 and Ago2. The EBV-encoded miRNAs, co-immunoprecipitated with Ago1 and Ago2, with reads of >10 000 counts (42 miRNAs) were plotted. (**d**) The ratio of sRNAs mapped to miRNA-registered loci, non-miRNA nonannotated loci, snRNA, snoRNA, mt-tRNA and the other noncording sRNA loci.

Notably, 30–40% of the total sRNAs in the latent-infected EBV cells were derived from the EBV genome, as was reported previously (4,11) (Figure 1a).

The majority of tags were mapped to regions annotated as both human and EBV miRNAs. Consistent with previous observations, the top 50 abundant sequence tags derived from the miRNA loci in both humans and EBV globally associate with Ago1 and Ago2 in approximately equivalent amounts (Figure 1b and c). Interestingly, tags that were derived from loci other than miRNA (non-miRNA loci) were found more often in the Ago1 than in the Ago2 IP sRNA library (26 versus 6%) (Figure 1d).

### A new class of sRNAs selectively associated with Ago1

**Figure 2.**
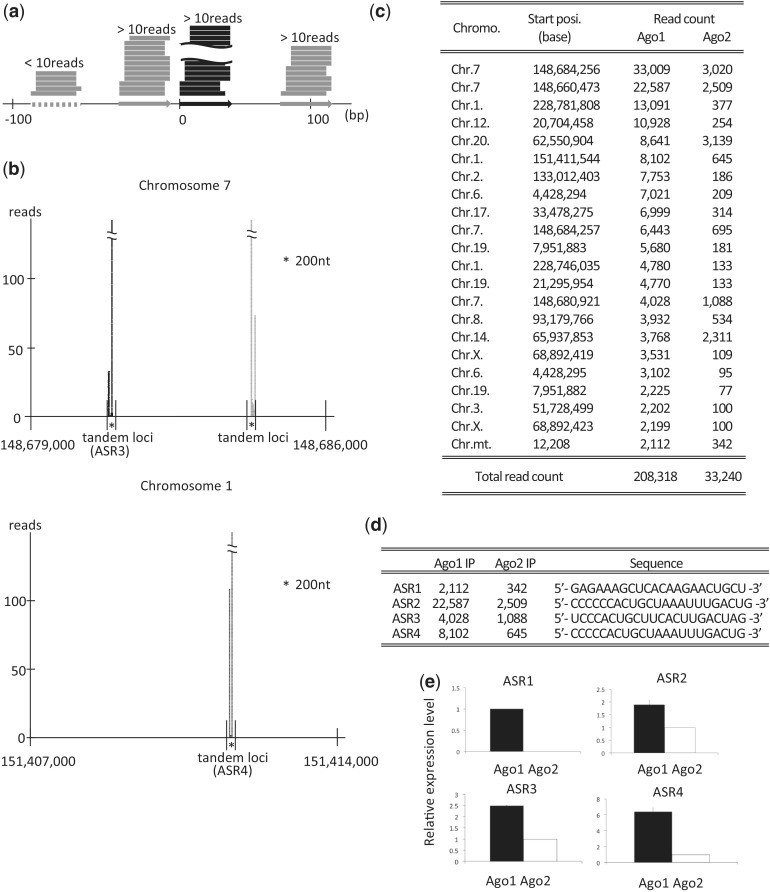
The sRNAs selectively associated with Ago1 are derived from tandem loci. (**a**) Definition of tandem loci and sRNA counts, which were mapped to the loci. When one locus (black arrow) with a read count of >10 reads and both flanking loci (gray arrows) located within 100 bases from the original locus also have >10 reads. (**b**) Representative loci are shown. Some Ago1 associating sRNAs are aligned to 228 776 000–228 783 000 bp area of chromosome 1 and 148 679 000–148 686 000 bp area of chromosome 7. The areas contain ASR3, ASR4 and many tandem locus. Black reads are aligned to forward strand, and gray reads to reverse strand. (**c**) Some unique sRNAs are selectively associated with Ago1. The counts of sRNAs associated with Ago1 and Ago2 (>2000 counts), derived from tandem loci, are listed. (**d**) The sequences of representative ASRs are shown. (**e**) Associations of ASR1, 2, 3 and 4 with Ago1 or Ago2 were determined by real-time PCR to validate the next-generation sequencing data. The data were normalized by the amount of RNA. Black bar indicates Ago1-associated RNA expression; white bar, Ago2.

Interestingly, some unique sequence tags from non-miRNA loci, derived from tandem loci in the human genome, predominantly associate with Ago1 but not, or only rarely, with Ago2. The definition of tandem loci is as follows: when one locus has a read count of >10, both flanking loci located within 100 bases from the first locus also have >10 reads (Figure 2a). The typical loci are shown in Figure 2b. More than 2000 counted RNAs are listed in Figure 2c. We named the sRNAs as Agotaxis small RNAs (ASRs). The representative ASRs were further investigated (Figure 2d). The results for several of these sRNAs by next-generation sequencing were validated by real-time PCR, which showed that the sRNA levels were significantly higher in Ago1- than Ago2-immunoprecipitated samples (Figure 2e). The expression of the sRNAs in L591 cells was confirmed by the northern blot analysis (12) (Supplementary Figure).

### Downregulation of DROSHA does not lead to the decrease of Agotaxis 2, 3 and 4 in L591 cells

**Figure 3.**
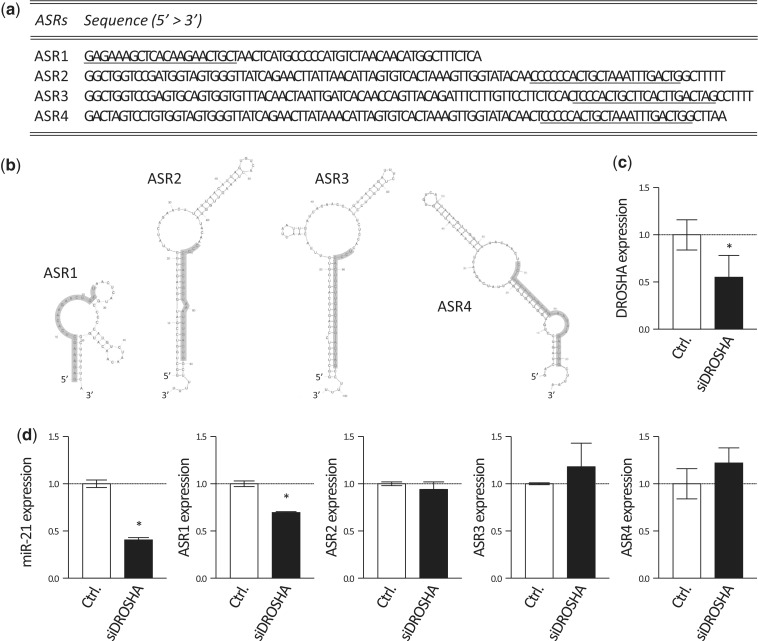
Processing of ASRs is independent of DROSHA. (**a**) Sequences of precursor of ASR transcripts are shown. That of ASR1 is ENST00000387449; ASR2, NR_004393.1; ASR2, NR_004392.1; ASR4, NG_032096.1. Underlined sequences indicate mature ASRs. (**b**) Structures of ASR1, 2, 3 and 4 were predicted with sequences indicated in Figure 3a by the mfold web server. Highlighted regions indicate mature ASRs sequences. (**c** and **d**) Control siRNA (Ctrl.) or siDROSHA were transfected into L591 cells by Neon™ transfection system. (c) Expressions of DROSHA was evaluated by real-time PCR 3 days after the transfection. Expression levels relative to Ctrl. were normalized with GAPDH and shown. (d) Cells were cultured for 6 days, and then total RNAs were extracted. Amounts of ASRs and mature miR-21 were determined by real-time PCR. Data were shown as relative expression to the Ctrl. normalized with RNU6. Error bars indicate SD. *P < 0.05.

From the registered sequence of ASRs transcripts shown in Figure 3a, we predicted their folding structures by the mfold web server (http://mfold.rna.albany.edu/?q=mfold/RNA-Folding-Form). Every ASR potentially form secondary hairpin loop structures (Figure 3b). We performed 5′ RACE and found only ASR1 transcript contained the flanking regions (data not shown). In the canonical pathway of miRNAs processing, the flanking regions of primary miRNAs are cleaved by DGCR8/DROSHA complex. We evaluated the expression of ASRs when DROSHA was knocked down in L591 cells by siRNA against DROSHA (siDROSHA). The expression of DROSHA was significantly decreased 3 days after the transfection (Figure 3c). The amounts of ASRs and mature miR-21 were evaluated with real-time PCR 6 days after the transfection (Figure 3d). While miR-21 was significantly decreased in siDROSHA-transfected L591 cells, the expressions of ASRs except ASR1 did not change (Figure 3d). About 30% reduction in ASR1 was confirmed (Figure 3d). The result suggested DROSHA is not involved in the processing of ASR2, 3 and 4.

### Characteristic features of the first nucleotide preference

**Figure 4.**
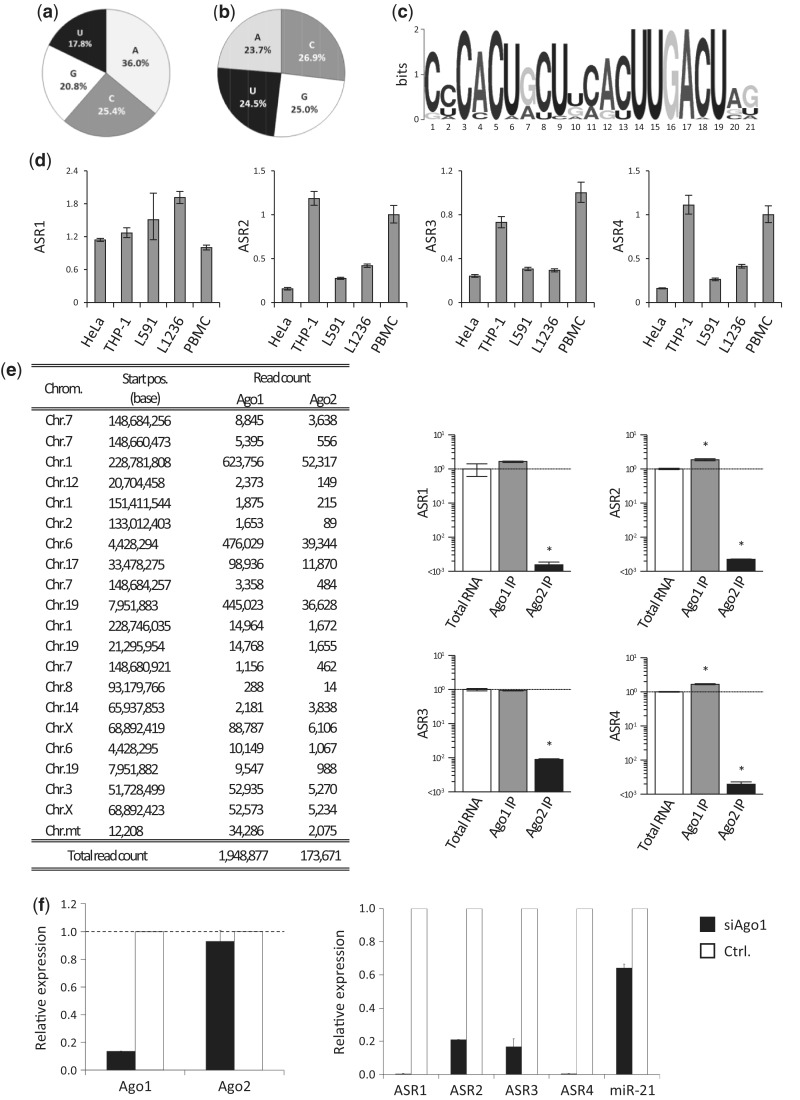
Characterization of ASRs. (**a**) The 5′ base of the top 500 abundantly expressed ASRs. (**b**) The composition of the top 500 abundantly expressed ASRs. (**c**) The consensus motif of top 500 abundantly expressed ASRs. (**d**) Expression of representative ASRs in HeLa, THP1, L591, L1236 and PBMCs were analyzed by real-time PCR, normalized by GAPDH (*n* = 3). (**e**) Associations of ASR1, 2, 3 and 4 with Ago1 or Ago2 in L1236 cells were determined by next-generation sequencing and real-time PCR normalized by the amount of RNAs. The read counts of the ASRs listed in Figure 2c in Ago1-IP and Ago2-IP L1236 cells are summarized. Error bars indicate SD. *P < 0.05. (**f**) mRNA expression of Ago1 and Ago2 in Ago1-knockdown (black bar) and control (white bar) L591 cells (left). Expression of representative ASRs under each condition. Both data were normalized by GAPDH.

The 5′ ends of miRNAs and piRNAs are mostly uridine, which likely binds to Ago/Piwi proteins (13). The 5′ ends of the top 500 abundantly expressed ASRs were analyzed. A large proportion of the 5′ ends of the ASRs are C, G or A (82.2%), with uridine being a minor component (17.8%). Therefore, these binding proteins are different from those associated with canonical miRNAs (Figure 4a). The composition of the nucleotides of ASRs is slightly biased to C [A (23.7%):G (25.0%):C (26.9%):U (24.5%); Figure 4b]. We investigated the position-specific motif for the top 500 abundantly expressed ASRs using the MEM software (http://meme.sdsc.edu). The top consensus motif of 37 ASRs from the 500 ASRs was CACU at the 5′ end +2 and UUGACU at the 3′ end (Figure 4c). The alignment of 37 ASRs was shown in Supplementary Figure S1. We observed substantial sequence variation in ASRs. Some of the isoforms of ASRs had comparable abundances with major ASRs (Figure 2c).

### Expression profiles of ASRs

Expression of ASRs in the peripheral blood mononuclear cell (PBMC), THP-1, HeLa and L1236 cells, which were derived from Hodgkin’s lymphoma cells but were not EBV infected, were analyzed. All cells and cell lines express ASRs. The expression of ASRs is not restricted to EBV-infected cell lines, but seemed to be ubiquitous (Figure 4d). Moreover, the expression of ASRs in Ago1 IP and Ago2 IP L1236 cells by use of the next-generation sequencing demonstrated that ASRs were selectively incorporated into Ago1, indicating that selective incorporation of these sRNAs into Ago1 is not restricted to cells infected by EBV (Figure 4e), which was again confirmed by the real-time PCRs (Figure4e).

### ASRs in cells are stabilized by Ago1 proteins

The expression of ASRs was not altered by RNase treatment after Ago1 IP, suggesting that ASRs bind specifically to Ago1 (data not shown). In addition, ASRs are highly dependent on Ago1. When Ago1 was knocked down, their expression decreased dramatically, while that of Ago2 was unchanged (Figure 4f left). In this condition, ASRs expression was determined by real-time PCR. In Figure 4f, right, the expression of miR-21, which is incorporated into Ago1 and Ago2 equally, was downregulated by half when Ago1 was knocked down. Expression of ASR2 and ASR3 in Ago1-knockdown cells was reduced to one-fifth that of the control. Most strikingly, the expression of ASR1 and ASR4 in Ago1-knockdown cells was reduced dramatically to almost below the detection limit. On the other hand, the amounts of ASRs did not change in mild Ago2 knocked down L591 cells (14) (Supplementary Figure S2). Free sRNAs are unstable in the cell and are digested rapidly (15). Therefore, ASRs are predominantly associated with Ago1 in a highly specific manner, which prevents ASRs incorporation by Ago2, 3 or 4. This was confirmed by determining the expression levels of sRNAs associated with Ago2 when Ago1 was knocked down. Expression levels of some were increased slightly in the Ago2 RISC complex, while others were unchanged (Supplementary Table S1). These results indicate that the majority of ASRs are incorporated preferentially into the Ago1–RISC complex.

### ASRs can silence targets, suggesting that ASRs are potentially functional

**Figure 5.**
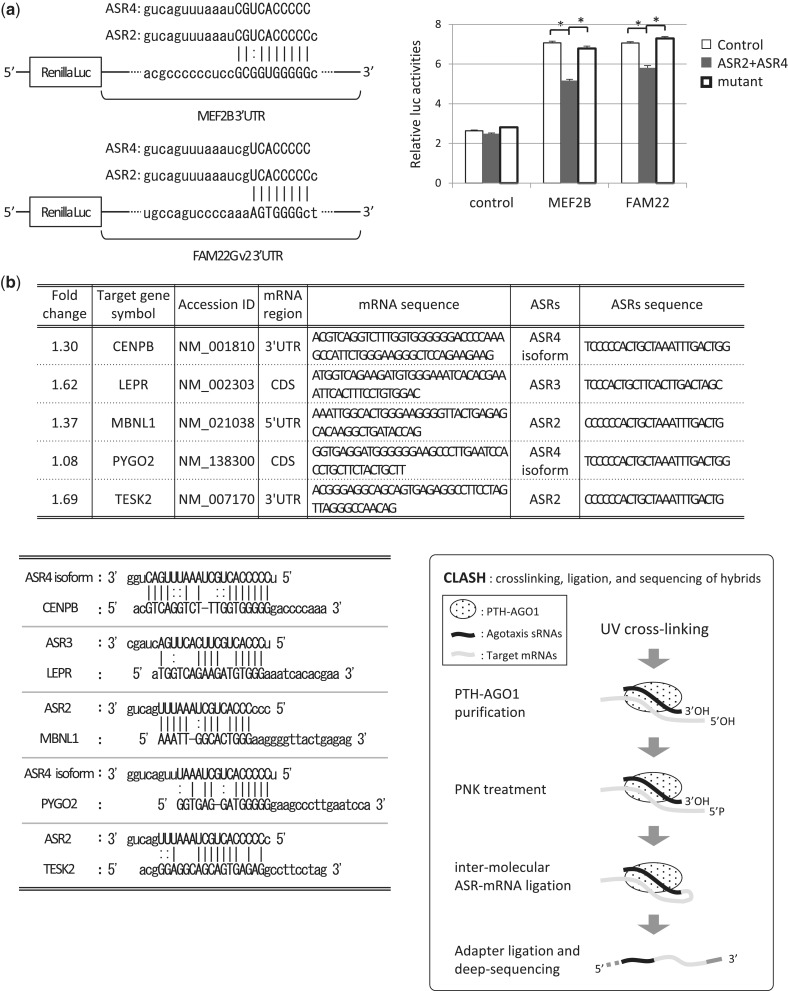
ASRs can bind and silence targets. (**a**) MEF2B and FAM 22 were regulated by ASR2 and ASR4 though their 3′ UTR. The putative target sequence of ASR2 and 4 in MEF2B and FAM22-3′ UTRs (left). Reporter vectors were constructed by inserting MEF2B and FAM22-3′ UTRs into Renilla luciferase in a psiCHECK2 vector. Renilla to firefly luciferase ratios are shown (right). All binding sites caused ASR2- and 4-dependent downregulation but not control or mutant (agggggacuaaauuugacgg) (*P < 0.05). (**b**) The sequence-specific binding of ASRs at the CDS, 3′ UTR and 5′ UTR of the target mRNAs in 293T cells, which were revealed by CLASH and downregulated in Ago1 knockdown L591 cells. The five genes, which have putative target sequences of ASRs in the mRNAs, were bound by ASRs in 293T cells, and upregulated by Ago1 knockdown in L591 cells are listed (upper). The putative target sequences of ASRs in the target mRNAs are shown (lower left). Overview diagrams of ASRs-mRNA chimera preparation (lower right). AGO1-associated sRNAs including ASRs and target mRNAs were cross-linked to PTH-AGO1 by UV exposure. The protein–RNA complexes were purified. mRNA 5′ ends were phosphorylated with PNK treatment and ligated to associated ASRs. Finally, 5′ and 3′ adapters were ligated to the chimeric RNAs for next-generation sequencing.

To determine their regulatory roles, we searched for putative targets of four abundantly incorporated ASRs and isoforms. *In silico* prediction of the binding sites of the 3′ untranslated region (UTR) of ASR2 and ASR4 by miRanda v3.3, which are highly downregulated in Ago1-knockdown L591 cells, are listed in Supplementary Figure S4. Among them, Family with sequence similarity 22 (FAM22) and Myocyte enhancer factor 2B (MEF2B) expression in Ago1-knockdown L591 cells was upregulated 2- and 4-fold, respectively (Supplementary Figure S4). ASR2 and ASR4 target sequences of FAM22 and MEF2B were shown in Figure 5a. The results were validated by assessment of the capacity to direct functional repression, using a luciferase assay.

We cloned the *FAM22* and *MEF2B* 3′ UTRs into a luciferase reporter vector and found that exogenous ASR2 and ASR4 repressed reporter activity, while the mutants had little or no effect (Figure 5a). This confirmed that ASR2 and ASR4 negatively regulate the mRNA expression of FAM22 and MEF2B directly through the 3′ UTR. The results indicate that these ASRs function in similar ways to miRNAs.

Moreover, the binding locus of ASRs in 293T cells was comprehensively investigated by use of the data sets previously reported (10). New technique for ligation and sequencing of miRNA-target RNA duplexes associated with human Ago1, known as ‘CLASH’, revealed that the majority of ASRs bind not only to mRNA 3′ UTRs but also to coding regions (CDS) and 5′ UTR (10). The library (∼18 000 high-confidence miRNA–mRNA interactions) contained the binding locus of top 20 abundantly expressed ASRs, which revealed 90 loci. Among them, centromere protein B (CENPB), leptin receptor (LEPR), Muscleblind-like splicing regulator 1 (MBNL1), pygopus family PHD finger 2 (PYGO2)and Testis-specific kinase 2 (TESK2), which have the putative target binding sites of ASRs, were upregulated in Ago1 knockdown L591 cells (Figure 5b). These results suggest that it is highly possible that these five genes are regulated by ASRs in L591 cells as well.

### Expression of ASRs in the EBV life cycle

We showed that many EBV-encoded miRNAs were expressed in EBV-latent-infected cells (Figure 1a). During acute EBV infection, the virus sequentially expresses its entire repertoire of genes, producing a lytic infection, while few genes are expressed during latent infection. Recently, it was reported that murine gammaherpesvirus 68 (MHV68) infection could induce and upregulate endo-siRNAs from short interspersed nuclear elements in murine cells during lytic infection (16). Therefore, linkage of the EBV life cycle with ASRs expression was investigated.

**Figure 6.**
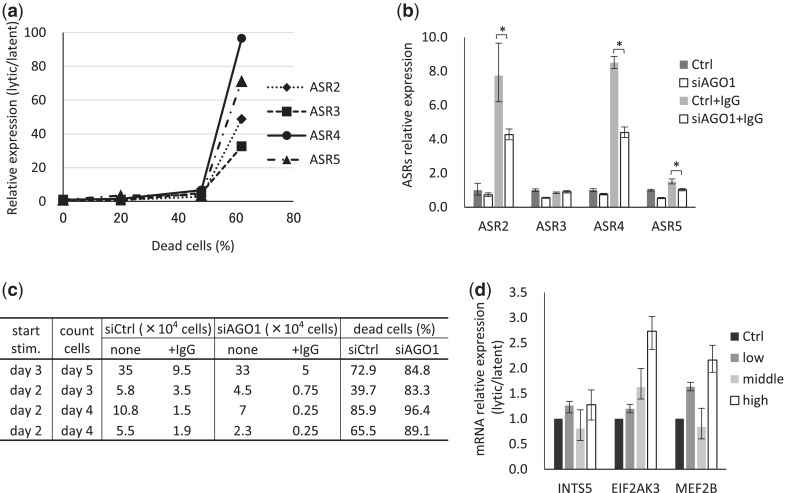
Expression of ASRs is correlated with the EBV life cycle. Akata cells were stimulated with anti-human IgG antibody and induced into the lytic phase. The cells were harvested at each point and total RNAs were purified. (**a** and **b**) The expression levels of total ASRs were measured by qPCR and calibrated with GAPDH. (a) ASRs relative expression levels are shown as fold-expression changes compared with that of the latent phase. (b) Akata cells were transfected with/without siRNA against AGO1 by Neon system (siAGO1 and Ctrl, respectively). After 2 days, cells were stimulated with/without anti-human IgG antibody to induce EBV lytic phase. After 2 more days in culture, cells were harvested and relative ASRs expression to Ctrl was measured by qPCR. Ctrl and siAgo1 indicate latent phase; Ctrl+IgG and siAgo1+IgG, lytic phase. *P < 0.05. (**c**) The cell numbers at harvested points were counted. The dead cell ratios were calculated by 1 − (IgG stimulated cell numbers/nonstimulated cell numbers). (**d**) The mRNA expression levels of ASRs’ target genes were calculated by qPCR and calibrated with GAPDH. Relative expression levels are shown as fold-expression changes compared with that of the latent phase. Ctrl indicates latent phase; low, <40%; middle, 40–60%; high, >60% dead cell ratio. The data are shown as means and SD.

Akata is a type 3 latent-infected cell line derived from an EBV-positive Burkitt’s lymphoma patient. The lytic phase was induced by Akata membrane IgG and anti-IgG cross-linking. When the lytic phase was induced, the cells underwent apoptosis. According to our results, expression level of ASR2, 3, 4 and 5 was linked with apoptotic cell numbers (Figure 6a). When the >60% Akata cells were dead, ASRs were increased >30-fold compared with latent-phase Akata cells. Moreover, when the lytic phase was induced with AGO1 knocked down cells, the expression of ASRs was decreased (Figure 6b). Under the condition, the apoptotic cell numbers tended to be upregulated (Figure 6c). These results indicated that in the lytic phase, the ASRs are significantly upregulated depending on AGO1.

The miRANDA predicted target genes of these sRNAs in L591 cells were listed (Supplementary Figure S4). Among them, we focused on INTS5, MEF2B and EIF2AK3, which are involved in viral reactivation, and analyzed their correlation with the EBV life cycle in Akata cells by qPCR (Figure 6d). The lytic phase was categorized by dead cell ratio into: low, <40%; middle, 40–60%; high, >60%. Expression of INTS5 and MEF2B showed a tendency to be decreased especially in middle level of the cell apoptosis. Interestingly, the expression of all these three genes tended to increase in high dead cell ratio (Figure 6d).

## DISCUSSION

In *Drosophila*, siRNAs and miRNAs are actively sorted into functionally distinct Ago-RISCs based on differences in structure (17,18). Perfectly matched duplexes are preferentially incorporated into Ago2, whereas duplexes with central mismatched bulges (miRNA-like) are sorted to Ago1 (17,18). A similar sorting mechanism exists in *Caenorhabditis elegans*, whereby sRNA duplexes with perfectly matched or bulged stems are channelled into RNAi-defective 1 (RDE-1) or Argonaute (plant)-like gene 1 (ALG-1), respectively (19).

In mammals, the regularity of miRNA sorting onto distinct Ago proteins is poorly understood. Mammals have four Agos (Ago1–4) that are involved in the miRNA pathway. Among them, Ago2 is unique and possesses the slicer activity that mediates the cleavage of perfectly matched targets for miRNAs and siRNAs (20). When individual Agos are constitutively ablated in mice, only the loss of Ago2 causes embryonic lethality, whereas single loss of Ago1, Ago3 or Ago4 is dispensable for animal development. However, RNA-sequencing of Ago1-, Ago2- and Ago3-associated miRNAs revealed that some have a bias toward particular Ago proteins (21).

We investigated the effect of viral infection on the sorting of sRNAs onto Ago proteins and identified sRNAs derived from the human genome and incorporated exclusively into Ago1 (ASRs).

The 5′ ends of the ASRs in are mostly C, G or A; uridine represents only a minor portion, implying that their binding proteins differ from those associated with canonical miRNAs (16).

In *Drosophila*, several studies have demonstrated that miRNA sorting onto Ago proteins is coupled to strand selection and depends on specific structural and sequence criteria, in which the sRNAs in the Ago1 protein complex with 5′ cytosine are more stable than those with 5′ uridine (13).The fate of a miRNA/miRNA* duplex, therefore, depends on multiple factors, including the structure of the duplex and the thermodynamic stability of the ends of the duplex (22). The importance of each factor in the sorting decision in mammals is as yet unknown.

We found that several ASRs harbor motifs that are preferred at locations on both their 5′ and 3′ ends.

The motifs on the 5′ ends of ASRs predict the target sequences that are commonly bound by ASRs. The common target sequence located in 3′ UTR of tumor suppressor genes has been recently reported, which is putatively bound by oncogenic miRNAs (23). The putative target sequence complementary to the motif on the 5′ ends of ASRs is completely different from the common target sequence bound by oncogenic miRNAs. It seems likely that the motif on the 5′ end of ASRs has some other regulatory functions.

The biogenesis of ASRs is independent from Drosha. They are incorporated in Ago1-RISC, bind mRNAs and work in similar way to miRNAs. Mirtrons also works as miRNA, which regulate RNA via a multistep process involving intron splicing and debranching, exosome-mediated trimming of the 3-tail and dicing. Top 20 highly expressed ASRs are coded within intergenic or transfer RNAs but not intron (24).

Accordingly, ASRs and Mirtron should be separately categorized. Still their biogenesis and functional mechanism seem to be overlapped. Further studies about the comparison of two kinds of sRNAs are needed.

Recently, 3′ motifs of miR-29 have been reported as nuclear transport signal (25). The 3′ common sequence of ASRs, which is not similar to the 3′ motif of miR-29, might be involved some other function such as transport and stability. Most importantly, whether both of the motifs are involved in the Ago1/Ago2 sorting decision needs further investigation.

ASRs regulate expression of their targets such as FAM22 and MEF2B by binding to the 3′ UTR in L591 cells. Therefore, ASRs have at least some functional similarity to miRNAs. Recent comprehensive analysis of sRNAs and their binding target RNAs, known ‘CLASH’, revealed that ASRs were incorporated into Ago1-RISC and bound to several mRNAs in 293T cells (10). Intriguingly, the data revealed that ASRs were also shown to bind mostly to CDS. Several genes, including CENPB, LEPR and PYGO2, bound by ASRs in Ago1-RISC in 293T cells had putative binding sites of ASRs and were upregulated by Ago1 knockdown in L591 cells, which suggest that these genes are highly possibly regulated by ASRs in L591 cells. CENPB, centromere protein B, is a DNA binding protein that recognizes CENP-B box in the centromeric alpha satellite DNA (26). The protein has an important role in assembly of kinetochore structure. In apoptotic cells, the protein is characterized as autoantigen that is cleaved to 60-kDa fragment and observed in blebs (27). It implies that ASRs may reduce autoimmune response in apoptotic phase.

By contrast, LEPR and PYGO2 are reported as anti-apoptotic elements. LEPR is known as the regulation of fat metabolism and hematopoiesis. Leptin activate Janus kinase/Signal transducer and activator of transcription (JAK/STAT) pathways via LEPR, which result in enhanced expression of anti-apoptotic factor, B-cell CLL/lymphoma 2 (BCL-2) (28). PYGO2 is a component of Wnt/beta-catenin transcriptional complex. Overexpression of PYGO2 reduces apoptotic activity in human carcinoma cell lines (29)

Together with our results in EBV lytic infection, ASRs might be involved in apoptosis. The significance and function of ASRs, including their binding to CDS warrants further investigation.

Finally, we analyzed the linkage of expression of ASRs with EBV infection status. Interestingly, the expression of ASRs was dramatically upregulated during lytic EBV infection. Small RNAs upregulations during lytic phase were consistent with the case of MHV68 infection (16). The gene expression pattern is dramatically changed between virus infectious latent and lytic phase. ASRs might be critical for this phase conversion. When Ago1 was knocked down, ASRs were downregulated and much more cells tended to be induced into apoptosis. ASRs depending on Ago1 might suppress apoptosis, terminal phase of lytic infection. The hypothesis was further evaluated by investigation of the target genes of ASRs involved in EBV reactivation. It is presumed that as more ASRs were expressed, there would be a corresponding decrease in the expression of the target genes. To the contrary, the general trend of the three genes supposed to be involved in EBV reactivation was upregulation correlatively to apoptosis levels (Figure 6d).

INTS5, integrator subunit 5, is a member of a multiprotein complex associated with the C-terminal repeats of RNA polymerase II, and mediates small nuclear RNA 3′-end processing (30). ASRs may reduce INTS5 function and suppress EBV-encoded miRNA processing. EIF2AK3, eukaryotic initiation factor 2-alpha kinase 3, is a type I endoplasmic reticulum (ER) transmembrane protein (31). It contains a stress-sensing domain. When unfolded proteins are sensed in the ER, protein synthesis is decreased, resulting in apoptosis (32). However, EIF2AK3 also has reported pro-tumorigenic properties (33). ASRs may function as a balancer of EIF2AK3 function, but further investigation is needed. MEF2B, myocyte enhancer factor 2B, is a transcriptional activator. A recent report showed that MEF2B (34) contributes to EBV-infected cell reactivation from the latent stage. Together with the upregulation of ASRs in the lytic infection, the result suggests that ASRs might have some functions through the posttranscriptional regulation of these target genes to prevent the lytic infection from ‘overactivation’.

In mammals, the Ago1–4 family sorting mechanism is unknown. Still, the class of sRNAs selectively incorporated onto Ago1, ‘ASRs’, appear to play vital roles in some aspects of mammalian biology. A mechanistic analysis of their processing and stability, and an extended investigation of the function of the ASRs in EBV-infected cells, needs thus further investigation.

## SUPPLEMENTARY DATA

Supplementary Data are available at NAR Online.

## FUNDING

Japan Society for the Promotion of Science, Takeda Science Fundation, Mitsui Life Social Walfare Fundation, Mochida Memorial Fundation for Medical and Pharmaceutical Research, Banyu Life Science Foundation International, International Myeloma Foundation Sugi Memorial (Awarded to A.K.) and Japan Leukemia Research Fund (Awarded to K.O.). Funding for open access charge: PRESTO/JST.

*Conflict of interest statement*. None declared.

## Supplementary Material

Supplementary Data

## References

[1] Bartel DP (2004). MicroRNAs: genomics, biogenesis, mechanism, and function. Cell.

[2] Deddouche S, Matt N, Budd A, Mueller S, Kemp C, Galiana-Arnoux D, Dostert C, Antoniewski C, Hoffmann JA, Imler JL (2008). The DExD/H-box helicase Dicer-2 mediates the induction of antiviral activity in drosophila. Nat. Immunol..

[3] Deleris A, Gallego-Bartolome J, Bao J, Kasschau KD, Carrington JC, Voinnet O (2006). Hierarchical action and inhibition of plant Dicer-like proteins in antiviral defense. Science.

[4] Skalsky RL, Corcoran DL, Gottwein E, Frank CL, Kang D, Hafner M, Nusbaum JD, Feederle R, Delecluse HJ, Luftig MA (2012). The viral and cellular microRNA targetome in lymphoblastoid cell lines. PLoS Pathog..

[5] Ghildiyal M, Xu J, Seitz H, Weng Z, Zamore PD (2010). Sorting of *Drosophila* small silencing RNAs partitions microRNA* strands into the RNA interference pathway. RNA.

[6] Su H, Trombly MI, Chen J, Wang X (2009). Essential and overlapping functions for mammalian Argonautes in microRNA silencing. Genes Dev..

[7] Van Stry M, Oguin TH, Cheloufi S, Vogel P, Watanabe M, Pillai MR, Dash P, Thomas PG, Hannon GJ, Bix M (2012). Enhanced susceptibility of Ago1/3 double-null mice to influenza A virus infection. J. Virol..

[8] Lu J, Chanda B, Kotani A (2012). Hematology - Science and Practice.

[9] Kube D, Holtick U, Vockerodt M, Ahmadi T, Haier B, Behrmann I, Heinrich PC, Diehl V, Tesch H (2001). STAT3 is constitutively activated in Hodgkin cell lines. Blood.

[10] Helwak A, Kudla G, Dudnakova T, Tollervey D (2013). Mapping the human miRNA interactome by CLASH reveals frequent noncanonical binding. Cell.

[11] Okuyama K, Ikawa T, Gentner B, Hozumi K, Harnprasopwat R, Lu J, Yamashita R, Ha D, Toyoshima T, Chanda B (2013). MicroRNA-126-mediated control of cell fate in B-cell myeloid progenitors as a potential alternative to transcriptional factors. Proc. Natl Acad. Sci. USA.

[12] Dueck A, Ziegler C, Eichner A, Berezikov E, Meister G (2012). microRNAs associated with the different human Argonaute proteins. Nucleic Acids Res..

[13] Li Z, Kim SW, Lin Y, Moore PS, Chang Y, John B (2009). Characterization of viral and human RNAs smaller than canonical MicroRNAs. J. Virol..

[14] Kosaka N, Iguchi H, Yoshioka Y, Takeshita F, Matsuki Y, Ochiya T (2010). Secretory mechanisms and intercellular transfer of microRNAs in living cells. J. Biol. Chem..

[15] Modzelewski AJ, Holmes RJ, Hilz S, Grimson A, Cohen PE (2012). AGO4 regulates entry into meiosis and influences silencing of sex chromosomes in the male mouse germline. Dev. Cell.

[16] Xia J, Zhang W (2012). Noncanonical microRNAs and endogenous siRNAs in lytic infection of murine gammaherpesvirus. PLoS One.

[17] Förstemann K, Horwich MD, Wee L, Tomari Y, Zamore PD (2007). *Drosophila* microRNAs are sorted into functionally distinct argonaute complexes after production by dicer-1. Cell.

[18] Tomari Y, Du T, Zamore PD (2007). Sorting of *Drosophila* small silencing RNAs. Cell.

[19] Steiner FA, Hoogstrate SW, Okihara KL, Thijssen KL, Ketting RF, Plasterk RH, Sijen T (2007). Structural features of small RNA precursors determine Argonaute loading in *Caenorhabditis elegans*. Nat. Struct. Mol. Biol..

[20] Liu X, Jin DY, McManus MT, Mourelatos Z (2012). Precursor microRNA-programmed silencing complex assembly pathways in mammals. Mol. Cell.

[21] Burroughs AM, Ando Y, de Hoon MJ, Tomaru Y, Suzuki H, Hayashizaki Y, Daub CO (2011). Deep-sequencing of human Argonaute-associated small RNAs provides insight into miRNA sorting and reveals Argonaute association with RNA fragments of diverse origin. RNA Biol..

[22] Gu S, Jin L, Zhang F, Huang Y, Grimm D, Rossi JJ, Kay MA (2011). Thermodynamic stability of small hairpin RNAs highly influences the loading process of different mammalian Argonautes. Proc. Natl Acad. Sci. USA.

[23] Hamilton MP, Rajapakshe K, Hartig SM, Reva B, McLellan MD, Kandoth C, Ding L, Zack TI, Gunaratne PH, Wheeler DA (2013). Identification of a pan-cancer oncogenic microRNA superfamily anchored by a central core seed motif. Nat. Commun..

[24] Ruby JG, Jan CH, Bartel DP (2007). Intronic microRNA precursors that bypass Drosha processing. Nature.

[25] Hwang HW, Wentzel EA, Mendell JT (2007). A hexanucleotide element directs microRNA nuclear import. Science.

[26] Cooke CA, Bernat RL, Earnshaw WC (1990). CENP-B: a major human centromere protein located beneath the kinetochore. J. Cell Biol..

[27] Ramírez-Sandoval R, Sánchez-Rodríguez SH, Herrera-van Oostdam D, Avalos-Díaz E, Herrera-Esparza R (2003). Antinuclear antibodies recognize cellular autoantigens driven by apoptosis. Joint Bone Spine.

[28] Skibola CF, Holly EA, Forrest MS, Hubbard A, Bracci PM, Skibola DR, Hegedus C, Smith MT (2004). Body mass index, leptin and leptin receptor polymorphisms, and non-hodgkin lymphoma. Cancer Epidemiol. Biomarkers Prev..

[29] De D, Chen A, Wu Z, Lv S, He G, Qi Y (2009). Overexpression of Pygopus2 protects HeLa cells from vinblastine-induced apoptosis. Biol. Chem..

[30] Baillat D, Hakimi MA, Näär AM, Shilatifard A, Cooch N, Shiekhattar R (2005). Integrator, a multiprotein mediator of small nuclear RNA processing, associates with the C-terminal repeat of RNA polymerase II. Cell.

[31] Atkins C, Liu Q, Minthorn E, Zhang SY, Figueroa DJ, Moss K, Stanley TB, Sanders B, Goetz A, Gaul N (2013). Characterization of a novel PERK kinase inhibitor with antitumor and antiangiogenic activity. Cancer Res..

[32] Su Q, Wang S, Gao HQ, Kazemi S, Harding HP, Ron D, Koromilas AE (2008). Modulation of the eukaryotic initiation factor 2 alpha-subunit kinase PERK by tyrosine phosphorylation. J. Biol. Chem..

[33] Bobrovnikova-Marjon E, Grigoriadou C, Pytel D, Zhang F, Ye J, Koumenis C, Cavener D, Diehl JA (2010). PERK promotes cancer cell proliferation and tumor growth by limiting oxidative DNA damage. Oncogene.

[34] Murata T, Narita Y, Sugimoto A, Kawashima D, Kanda T, Tsurumi T (2013). Contribution of myocyte enhancer factor 2 (MEF2) family transcription factors to BZLF1 expression in Epstein-Barr virus reactivation from latency. J. Virol..

