# Supplementary material for: Novel functional small RNAs are selectively loaded onto mammalian Ago1
Source: Nucleic Acids Res. 2014 Mar 13;42(8):5289–301. doi: 10.1093/nar/gku137 (PMC4005649; doi:10.1093/nar/gku137)
Supplement: Supplementary Data [file supp_42_8_5289__index.html]

Novel functional small RNAs are selectively loaded onto mammalian Ago1 — Novel functional small RNAs are selectively loaded onto mammalian Ago1 — Supplementary Data 

# Novel functional small RNAs are selectively loaded onto mammalian Ago1

## Supplementary Data

files

**Files in this Data Supplement:**

- Supplementary Data - pdf file
