# Supplementary material for: Novel functional small RNAs are selectively loaded onto mammalian Ago1
Source: Nucleic Acids Res. 2014 Mar 13;42(8):5289–301. doi: 10.1093/nar/gku137 (PMC4005649; doi:10.1093/nar/gku137)
Supplement: Supplementary Data [file supp_gku137_nar-02493-y-2013-File009.pdf]

Supplemental Table1

experiment	Target	sequence
siRNA for knock down	*Ago1*	5'-gagaagaggugcucaagaauu-3'
	Ago2	5'-gcaggacaaagauguauuauu-3'
	DROSHA	5'-aaccgaagaucaccaucucuguu-3'
Quantitative PCR	miR-21	forward: 5'-tagcttatcagactgatgttg-3'
	miR-155	forward: 5'-ttaatgctaatcgtgataggggt-3'
	RNU6	forward: 5'-cgcaaggatgacacgcaaattc-3'
	ASR1	forward: 5'-gagaaagctcacaagaactgc-3'
	ASR2	forward: 5'-ccccccactgctaaatttgac-3'
	ASR3	forward: 5'-tcccactgcttcacttgacta-3'
	ASR4	forward: 5'-cccccactgctaaatttgact-3'
	ASR5	forward: 5'-aagcagggtcgggcctggtta-3'
	GAPDH	forward: 5'-ccactcctccacctttgac-3'<br>reverse: 5'-accctgttgctgtagcca-3'
	Ago1	forward: 5'-tagcaaattgatgcgaagtg-3'<br>reverse: 5'-caccaaagatctgaggcttg-3'
	Ago2	forward: 5'-tagcaaattgatgcgaagtg-3'<br>reverse: 5'-gcaatagctttattcctgcc-3'
	DROSHA	forward: 5'-tcaccatctctggaaaggtc-3'<br>reverse: 5'-caaccgataaaccgtaactc-3'
	INTS5	forward: 5'-aggggacgtagccttttcttg-3'<br>reverse: 5'-agcgctctttagaccaaggac-3'
	EIF2AK3	forward: 5'-ctgactacatatggactcagtgc-3'<br>reverse: 5'- ctgtagaagcaggatgtcttcc-3'
	MEF2B	forward: 5'- aaatccagatctcccgcatcc-3'<br>reverse: 5'- tggcgctgttgaagatgatg-3'
siRNA for dual luciferase assay	ASR2	5'-cccccacugcuaaauuugacug(dTdT) -3'
	ASR4	5'-ucccccacugcuaaauuugacugg(dTdT)-3'
	Mutant	5'-agggggacuaaauuugacgg(dTdT)-3'
	Control	5'-ccuacgccaccaauuucgu(dTdT)-3'

Supplemental Table 2

	L591<br>Ago2 IP (Ct)	L591kd<br>Ago2 incorporation
ASR1	undetected	undetected
ASR2	23.07	—
ASR3	29.81	↑
ASR4	24.43	—
miR-21	19.91	↑

Incorporation change of ASR-1-4 and mir-21 into Ago2 in Ago1 knocked down cell
(-) : unchanged (↑) : up regulated

Supplemental figure 1

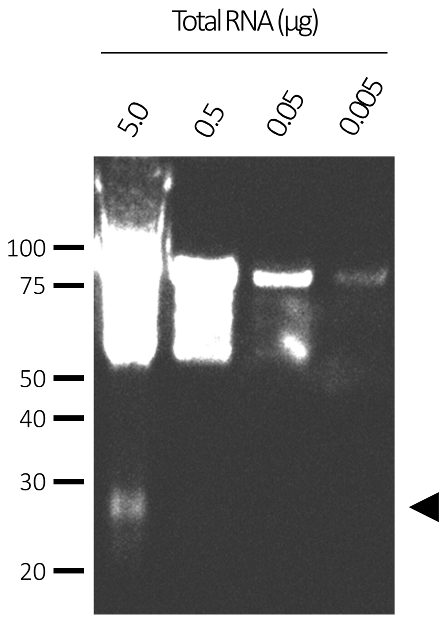

Expressions of ASR3 in L591 cells were confirmed by Northern blotting. Arrowhead indicates the mature ASR3. Serially diluted total RNAs (5.0, 0.5, 0.05, and 0.005 μg) were applied for Northern blotting.

Supplemental figure 2

Name	Strand	Start	*p*-value	Sites
chr20.fa_R_16651312	+	1	2.32e-13	CCCACUGCUUCACUUGACUAG UCU
chr16.fa_R_489323	+	1	2.32e-13	CCCACUGCUUCACUUGACUAG CCA
chr7.fa_F_148680922	+	1	2.32e-13	CCCACUGCUUCACUUGACUAG CCU
chr9.fa_R_21160261	+	2	2.32e-13	U CCCACUGCUUCACUUGACUAG AU
chr20.fa_R_16651313	+	2	2.32e-13	U CCCACUGCUUCACUUGACUAG UC
chr9.fa_R_21160263	+	4	2.32e-13	ACU CCCACUGCUUCACUUGACUAG
chr9.fa_R_21160262	+	3	2.32e-13	CU CCCACUGCUUCACUUGACUAG A
chr20.fa_R_16651314	+	3	2.32e-13	CU CCCACUGCUUCACUUGACUAG U
chr7.fa_F_148680920	+	3	2.32e-13	CU CCCACUGCUUCACUUGACUAG C
chr7.fa_F_148680921	+	2	2.32e-13	U CCCACUGCUUCACUUGACUAG CC
chr19.fa_R_6572994	+	2	1.35e-12	U CCCACUGCUUCACUUGACUAU CC
chr19.fa_R_6572995	+	3	1.35e-12	CU CCCACUGCUUCACUUGACUAU C
chr19.fa_R_6572996	+	4	1.35e-12	ACU CCCACUGCUUCACUUGACUAU
chr3.fa_R_38546999	+	3	2.06e-12	CU CCCACUGCUUCACUUGACUGG C
chr3.fa_R_38547000	+	4	2.06e-12	ACU CCCACUGCUUCACUUGACUGG
chr5.fa_R_78466084	+	2	3.22e-12	U CCCACUGCUUCACUUGACUAA CC
chr5.fa_R_78466085	+	3	3.22e-12	CU CCCACUGCUUCACUUGACUAA C
chr5.fa_R_78466086	+	4	3.22e-12	ACU CCCACUGCUUCACUUGACUAA
chr7.fa_R_148684254	+	1	8.91e-12	CUCACUACUGCACUUGACUAG UCU
chr7.fa_R_148684255	+	2	8.91e-12	U CUCACUACUGCACUUGACUAG UC
chr7.fa_R_148684257	+	4	8.91e-12	CUU CUCACUACUGCACUUGACUAG
chr5.fa_R_17117713	+	3	8.91e-12	UU CUCACUACUGCACUUGACUAG C
chr1.fa_R_156453918	+	3	8.91e-12	UU CUCACUACUGCACUUGACUAG G
chr7.fa_R_148684256	+	3	8.91e-12	UU CUCACUACUGCACUUGACUAG U
chr4.fa_R_79553455	+	3	2.05e-10	UU CUCACUACUGCACUUGACUCA U
chr4.fa_R_79553456	+	4	2.05e-10	CUU CUCACUACUGCACUUGACUCA
chr7.fa_F_148660476	+	1	2.56e-10	CCCACUGCUAAAUUUGACUGG CUU
chr7.fa_F_148660475	+	2	2.56e-10	C CCCACUGCUAAAUUUGACUGG CU
chr7.fa_F_148660474	+	3	2.56e-10	CC CCCACUGCUAAAUUUGACUGG C
chr7.fa_F_148660473	+	4	2.56e-10	CCC CCCACUGCUAAAUUUGACUGG
chr7.fa_F_148638630	+	3	1.60e-09	CC CCCACAACCGCGCUUGACUAG C
chr7.fa_F_148638629	+	4	1.60e-09	CCC CCCACAACCGCGCUUGACUAG
chr13.fa_R_50482799	+	4	2.10e-09	CCC CCCACUGCUAAAUUUGAAUGG
chr8.fa_R_70602371	+	1	1.25e-08	GACCCUGUUGAGCUUGACUCU AGU
chr8.fa_R_70602373	+	3	1.25e-08	AA GACCCUGUUGAGCUUGACUCU A
chr1.fa_F_237766555	+	4	1.25e-08	GAA GACCCUGUUGAGCUUGACUCU
chr1.fa_F_237766557	+	2	1.25e-08	A GACCCUGUUGAGCUUGACUCU AG

It is each sequences of the consensus motif (Figure 4c). The motif indicated significantly low E-value (1.9e-218). The consensus motif was analysed with following options: -dna -time 18000 -maxsize 60000 -mod zoops -nmotifs 3 -minw 6 -maxw 50 –revcomp.

Supplemental figure 3

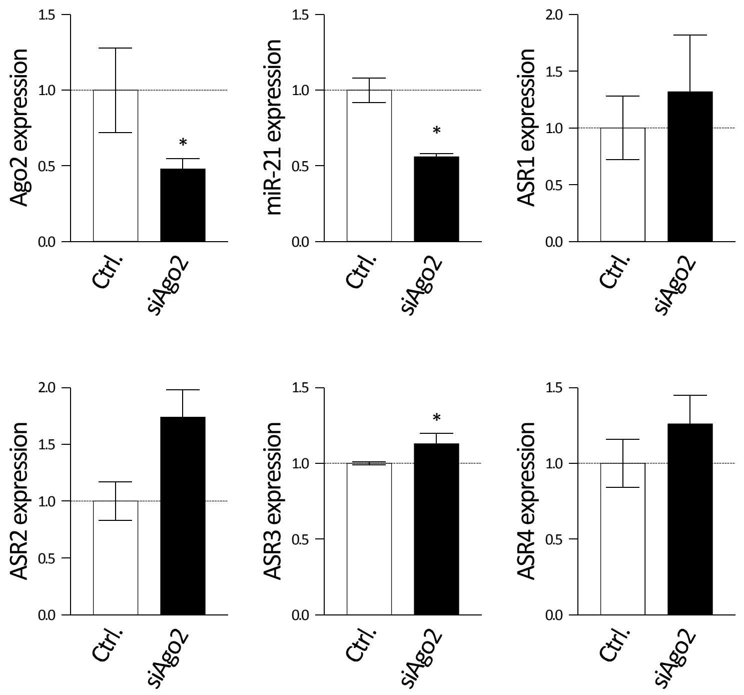

Control siRNA (Ctrl.) or siRNA targeting Ago2 (siAgo2) were transfected into L591 cells by Neon™ transfection system. Expression of Ago2 was determined 3 days after the transfection. Relative expression of miR-21, ASR1, 2, 3, and 4 were evaluated by real-time PCR 6, days after the transfection. Data were normalized with GAPDH (for Ago2) or RNU6 (for miR-21 and ASRs). Error bars indicate SD. *: *p*-value < 0.05

Supplemental figure 4

Representative target genes	Functional annotation
SCN5A	Voltage-gated sodium channel activity
MEF2B	Sequence-specific DNA binding, transcription factor activity
FAM22G	unknown
FCGBP	Protein binding
BRPF3	Protein binding, metal ion binding, zinc ion binding
ZNF853	Nucleic acid binding, metal ion binding
TOLLIP	Signal transduction (through IL1R, Toll-Like Receptor Pathway, Toll-like receptor signaling pathway), innate immunit
TTBK1	ATP binding, nucleotide binding, protein serine/threonine kinase activity, transferase activity
ELFN2	Protein phosphatase inhibitor activity, phosphatase binding
INTS5	Protein binding, snRNA processing
GBP1	GTP binding, GTPase activity, nucleotide binding
EIF2AK3	Activation of caspase activity, ATP binding, nucleotide binding, protein serine/threonine kinase activity, transferase activity

(b)

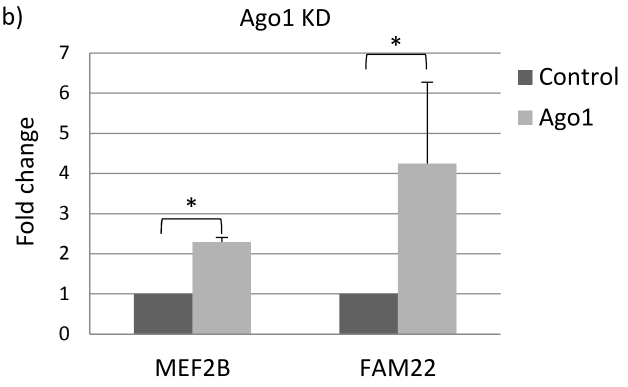

Representative predicted target genes of ASR2 and ASR4 by miLANDAare shown in the table. (left)
Microarray data represents that among the target genes, MEF2B and FAM22 are highly upregulated in Ago1 -knockdown condition compared with control in L591 cells. (* indicates $p < 0.05$)(right)
